# Supplementary material for: Lamellipodin promotes actin assembly by clustering Ena/VASP proteins and tethering them to actin filaments
Source: eLife. 2015 Aug 21;4:e06585. doi: 10.7554/eLife.06585 (PMC4543927; doi:10.7554/eLife.06585)
Supplement: Supplementary file 1. — Table of plasmid DNA used for protein expression and cellular transfections. DOI: http://dx.doi.org/10.7554/eLife.06585.030 [file elife06585s001.doc]

| **plasmid ID#** | **XTC/B16F1 cell expression** | **marker** |
| --- | --- | --- |
| **pSH247** | pDeltaCMV-EGFP-Lpd (1-1250aa) | KAN |
| **pSH633** | pCMV-EGFP-Lpd (1-1250aa) | KAN |
| **pSH394** | Lpd (850-1250aa) 44RK -> 44A (Invitrogen synthesized clone) | KAN |
| **pSH280** | pDeltaCMV-Lyn_11_-EGFP | KAN |
| **pSH307** | pDeltaCMV-Lyn_11_-EGFP-Lpd (850-1250) | KAN |
| **pSH328** | pDeltaCMV-Lyn_11_-EGFP-Lpd (850-1250) + (AAPPP)x6 | KAN |
| **pSH395** | pDeltaCMV-Lyn_11_-EGFP-Lpd (850-1250) + (44RK-44A) | KAN |
| **pSH601** | pDeltaCMV-Lyn_11_-EGFP-Lpd (850-1250) + SH3* | KAN |
| **pSH608** | pDeltaCMV-Lyn_11_-EGFP-Lpd (850-1250) + (AAPPP)x6 + SH3* | KAN |
| **pSH613** | pDeltaCMV-Lyn_11_-EGFP-Lpd (850-1250) + (44RK -> 35A) actin binding mutant | KAN |
| **pSH609** | pCMV-EGFP-Lpd (850-1250aa) | KAN |
| **pSH625** | pCMV-EGFP-Lpd (850-1250aa) + (AAPPP)x6 | KAN |
| **pSH626** | pCMV-EGFP-Lpd (850-1250aa) + SH3* | KAN |
| **pSH627** | pCMV-EGFP-Lpd (850-1250aa) + (AAPPP)x6 + SH3* | KAN |
| **pSH628** | pCMV-EGFP-Lpd (850-1250aa) + (44RK -> 35A) actin binding mutant | KAN |
| **pSH610** | pCMV-EGFP-LZ-Lpd (850-1250aa) | KAN |
| **pSH629** | pCMV-EGFP-LZ-Lpd (850-1250aa) + (AAPPP)x6 | KAN |
| **pSH630** | pCMV-EGFP-LZ-Lpd (850-1250aa) + SH3* | KAN |
| **pSH631** | pCMV-EGFP-LZ-Lpd (850-1250aa) + (AAPPP)x6 + SH3* | KAN |
| **pSH632** | pCMV-EGFP-LZ-Lpd (850-1250aa) + (44RK -> 35A) actin binding mutant | KAN |
| **pSH369** | pDeltaCMV-mCherry-Actin (human beta) | KAN |
| **pSH602** | pDeltaCMV-EGFP-PLCd | KAN |
|  |  |  |
|  | **bacterial protein expression** |  |
| **pSH181** | his10-TEV-EGFP-Lpd (850-1250aa) | KAN |
| **pSH291** | his10-TEV-EGFP-Lpd (850-1250aa) + (AAPPP)x6 | KAN |
| **pSH338** | his6-Z tag-EGFP-GGG-LZ-GGG-Lpd (850-1250aa) | KAN |
| **pSH402** | his10-TEV-EGFP-Lpd (850-1250aa) + (44RK -> 44A) actin binding mutant | KAN |
| **pSH179** | his6-TEV-KCK-VASP (1-114aa, human), EVH1 domain, Cys-light (C7S, C64S) | AMP |
| **pSH82** | his6-TEV-KCK-VASP (1-380aa, human) Cys-light (C7S, C64S, C334A) | AMP |
| **pSH140** | his6-TEV-KCK-EVL (1-393aa, mouse) Cys-Light (C7S, C177S) | AMP |
| **pSH196** | his6-TEV-KCK-VASP (1-380aa; L226A, I230A, L235A, R273A, R274A, R275A, K276A) | AMP |
| **pSH109** | his6-TEV-KCK-VASP (1-380aa; R273E, R274E, R275E, K276E) | AMP |
|  |  |  |
|  | **abbreviations** |  |
|  | **LZ**, Leucine zipper; **(AAPPP)x6**, Ena/VASP protein binding mutant; **Lpd,** Lamellipodin |  |
|  | **SH3***, mutations in Abi1/Endophilin SH3 domain binding sites; **CMV,** Cytomegalovirus promoter |  |
